# Wobble Board Instability Enhances Compensatory CoP Responses to CoM Movement Across Timescales

**DOI:** 10.3390/s25144454

**Published:** 2025-07-17

**Authors:** Mahsa Barfi, Theodoros Deligiannis, Brian Schlattmann, Karl M. Newell, Madhur Mangalam

**Affiliations:** 1 Department of Biomechanics, University of Nebraska at Omaha, Omaha, NE 68182, USA; 2 Department of Kinesiology, University of Georgia Athens, Athens, GA 30602, USA

**Keywords:** balance, center of mass, center of pressure, coordination, fractal regression, postural sway

## Abstract

This study investigated the interplay of bodily degrees of freedom (DoFs) governing the collective variable comprising the center of pressure (CoP) and center of mass (CoM) in postural control through the analytical lens of multiplicative interactions across scales. We employed a task combination involving a wobble board, introducing mechanical instability mainly along the mediolateral (ML) axis and the Trail Making Task (TMT), which imposes precise visual demands primarily along the anteroposterior (AP) axis. Using Multiscale Regression Analysis (MRA), a novel analytical method rooted in Detrended Fluctuation Analysis (DFA), we scrutinized CoP-to-CoM and CoM-to-CoP effects across multiple timescales ranging from 100ms to 10s. CoP was computed from ground reaction forces recorded via a force plate, and CoM was derived from full-body 3D motion capture using a biomechanical model. We found that the wobble board attenuated CoM-to-CoP effects across timescales ranging from 100to400ms. Further analysis revealed nuanced changes: while there was an overall reduction, this encompassed an accentuation of CoM-to-CoP effects along the AP axis and a decrease along the ML axis. Importantly, these alterations in CoP’s responses to CoM movements outweighed any nonsignificant effects attributable to the TMT. CoM exhibited no sensitivity to CoP movements, regardless of the visual and mechanical task demands. In addition to identifying the characteristic timescales associated with bodily DoFs in facilitating upright posture, our findings underscore the critical significance of directionally challenging biomechanical constraints, particularly evident in the amplification of CoP-to-CoM effects along the AP axis in response to ML instability. These results underscore the potential of wobble board training to enhance the coordinative and compensatory responses of bodily DoFs to the shifting CoM by prompting appropriate adjustments in CoP, thereby suggesting their application for reinstating healthy CoM–CoP dynamics in clinical populations with postural deficits.

## 1. Introduction

### 1.1. Overview of Postural Control

Human standing posture is an intricate dynamic process characterized by continuous subtle movements across limb segments and joints orchestrated to counteract the constant force of gravity. This orchestration aims to uphold the motion of the postural center of mass (CoM) within the stable confines of the base of support (BoS) [1,2,3]. The CoM, representing the body’s collective mass, is a pivotal reference point in postural control. Equilibrium demands alignment of the CoM with the base of support, typically the area beneath the body in contact with the supporting surface (e.g., the feet during standing). Any deviation of the CoM beyond this support base engenders a moment or torque around the support area, necessitating muscular intervention to preserve balance. For instance, a forward lean displaces the CoM ahead of the feet, triggering a muscular response to counteract the destabilizing moment and avert a fall. On the other hand, the postural center of pressure (CoP) designates where the ground reaction force vector—exerted by the ground on the body—is applied to the supporting surface. Unlike a static marker, the CoP is dynamically influenced by the body’s CoM and external forces acting upon it. Emerging research highlights the significance of understanding the joint dynamics of CoM and CoP in elucidating postural adaptations under diverse task constraints [4,5,6,7,8,9]—which is essential to devise interventions to reduce falls.

### 1.2. Challenges in Understanding CoM–CoP Coupling

Despite recent advancements, the interplay between CoM and CoP remains poorly understood. It remains elusive whether alterations in one invariably influence the other or if a causal relationship exists, with one variable dynamically responding to changes in the other. This constraint stems from the broader challenge posed by the inherent complexity of motor coordination, control, and skill execution [10,11,12]. These processes involve a bodywide movement system characterized by numerous redundant degrees of freedom (DoFs)—that is, the many ways in which the body’s joints, muscles, and neural pathways can contribute to a given movement—spanning the neural–muscular–articular level. Addressing this knowledge gap is paramount, particularly in discerning potential deficits contributing to balance impairments in aging populations and those afflicted with neurodegenerative conditions associated with postural deficits. A comprehensive understanding of these relationships holds promise for informing targeted interventions to mitigate postural deficits in clinical settings.

### 1.3. CoM–CoP as a Collective Variable

A longstanding debate in postural control concerns whether CoP motion results from a simple linear inverted pendulum model stabilizing the CoM or from a more complex, nonlinear, multi-linkage system [1,2,3,13,14,15]. If body sway were governed solely by the ankle joint acting like a pendulum, then local synergy—specifically, ankle joint movement—would explain most variations in CoP. However, accumulating evidence contradicts this prediction, indicating that intermediate-level neural–muscular–articular synergies coordinate CoM and CoP through distributed control involving multiple body segments [4,5,6,7,8,9,16,17,18,19,20,21,22,23,24,25,26,27].

From a dynamical systems perspective, such coordination is often described in terms of a *collective variable* (or order parameter)—a low-dimensional descriptor that captures the emergent behavior of many interacting components. In this context, the CoM–CoP relationship serves as a collective variable because it summarizes the global state of the postural system arising from the coordinated actions of joints, muscles, and neural processes. Rather than tracking every local movement, the nervous system may instead regulate this emergent variable to maintain an upright stance.

CoM represents the weighted spatial average of the body’s mass distribution, while CoP marks the location of the resultant ground reaction force. Together, their interaction reflects how the body uses ground reaction forces to stabilize the moving mass above. Within this framework, the CoM–CoP dynamic is not merely the outcome of joint-level control but a higher-order variable shaped by the self-organizing coordination of multiple DoFs. This view challenges the notion that posture is maintained by fixed coordination patterns and instead suggests that the nervous system flexibly tunes CoM–CoP coupling to meet task demands. Such tuning may differ across timescales, directions, and task constraints, highlighting the adaptive nature of postural control as a multiscale, context-sensitive process.

### 1.4. Postural Control Across Multiple Scales

While the dynamical systems approach has adeptly identified the CoM–CoP dynamics as a collective variable facilitating postural adaptations, the precise interactions between CoM and CoP remain elusive. The CoM–CoP dynamics must reflect various processes unfolding across multiple spatial and temporal scales [28,29,30,31]. For example, the seemingly straightforward task of maintaining a stationary stance while engaging in a secondary visual task masks a complex interplay of processes across various scales. At fine scales, success hinges upon the precise coordination of a few photoreceptors on the retina to encompass the entirety of the target within the visual field [32,33]. This foundational act, however, is intricately intertwined with coarser-scale factors such as the structural integrity of the surface beneath and the perceptible flow of visual stimuli [34,35]. Between these macroscopic elements, standing erect and engaging visually involves synergistic engagement across limbs and joints, orchestrating subtle adjustments in posture that elicit reflex responses and minor muscle contractions. This intricate coordination intertwines activities across multiple spatial and temporal scales, forging a tight nexus between phenomena spanning from macroscopic postural shifts to coarse-grained head and eye movements down to the fine-grained photoreceptor activation. The intricacy of this tapestry becomes most apparent in its clinical absence, particularly in populations confronting peripheral disruptions from *neural–muscular–articular* disorders and diseases affecting the central nervous system (CNS) [36,37,38,39,40,41], highlighting the inherently multiscale nature of postural control and the need to characterize coordination patterns that emerge from interactions spanning multiple spatial and temporal levels. To address these complexities, we turn to a method designed to resolve directional relationships across multiple timescales.

### 1.5. Motivation for Multiscale Analysis

To rigorously investigate the multiscale interactions between CoM and CoP, we require an analytical framework capable of capturing nonlinear, scale-dependent relationships. In that case, the influence of CoM on CoP transcends a singular timescale. Unlike prevailing linear models that typically account for control within a single timescale, this nonlinear influence necessitates consideration across various timescales. In the parlance of neurophysiology, the coordination compensatory activity of bodily DoFs span extremely short durations indicative of mechanotransduction, spanning from sub-millisecond to a few milliseconds [42,43], medium timescales associated with short-latency reflexes (SLRs, 20–50ms) and long-latency reflexes (LLRs, 50–100ms) [44,45,46,47,48,49], and longer timescales corresponding to compensatory postural adjustments (CPAs; >100 ms) [50,51,52,53,54]. Then again, postural control interweaves these processes into an adaptive whole characterized by distinct CoM–CoP dynamics that unfold across multiple scales [55]. This suggests that the CoM–CoP relationship could vary depending on the measurement scale. Thus far, analytically probing such multiscale relationships between biomechanical measurements like CoM and CoP has presented significant challenges. Fortunately, a cutting-edge method called Multiscale Regression Analysis (MRA) [56,57,58] offers a powerful analytical method for examining relationships between two variables across different scales. This approach allows researchers to capture and interpret the intricate interactions and dependencies inherent in data exhibiting multiscale variability. In the context of CoM–CoP dynamics, MRA might help illuminate how postural destabilizing constraints influence the relationship between CoM–CoP dynamics across multiple scales and provide insights into the coordinative and compensatory activity of bodily DoFs.

### 1.6. Directionality of CoM–CoP Coupling

Importantly, the directionality of CoM–CoP interactions has distinct control implications [3,52]. CoP-to-CoM effects are typically associated with feedforward mechanisms, reflecting anticipatory adjustments in pressure that help guide or stabilize the CoM before it destabilizes. In contrast, CoM-to-CoP effects are more often interpreted as feedback responses, where the system reacts to ongoing CoM movements with pressure shifts to restore balance. Disentangling these directional relationships is essential for understanding how the body flexibly organizes compensatory strategies under varying task constraints. Evaluating these effects across multiple timescales can reveal how proactive and reactive control processes unfold in parallel to support dynamic postural stability.

### 1.7. Study Objectives and Hypotheses

In the present study, we investigated how the coordinative and compensatory activity of bodily DoFs govern the collective variable of CoM–CoP dynamics from the lens of multiplicative interactions across scales. To quantify how the relationship between CoM and CoP evolves across timescales, we employed two complementary time-series techniques. Detrended Fluctuation Analysis (DFA) allows us to detect long-range correlations within a single signal, while Multiscale Regression Analysis (MRA) extends this approach to quantify directional coupling between two signals—such as how CoM influences CoP—across multiple temporal scales. We employed a wobble board featuring a rectangular plate affixed to three parallel semi-cylindrical components, inducing a “roll” instability along the anatomical ML axis. Our objective was to ascertain how postural instability along the ML axis reshapes the collective variable of CoM–CoP dynamics. We tested the central hypothesis that if postural instability due to the wobble board heightens CoP’s responses to CoM changes, we should observe an accentuation of CoM-to-CoP effects across timescales attributable to the coordinative and compensatory activity of bodily DoFs. We refined this central hypothesis into three specific hypotheses for a thorough evaluation: the first two are independent of the direction of postural sway, while the third tests predictions related to the AP and ML axes.

**Hypothesis** **1.** *We first hypothesized that the challenging mechanical constraints inherent in stabilizing the wobble board would accentuate CoP’s responses to CoM changes indicative of heightened coordinative and compensatory activity of bodily DoFs at timescales specific to neural–muscular–articular synergies (i.e., 100–400 ms). Conversely, as part of our control, we anticipate that CoM will not exhibit sensitivity to CoP alterations across these timescales, suggesting a unidirectional relationship between CoM and CoP.*

**Hypothesis** **2.** *While it might seem intuitive that lateral (ML-tending) “roll” perturbations would elicit coordinative and compensatory activity of bodily DoFs in the same direction along the ML axis, biomechanical constraints dictate that the upright postural response to “roll” perturbation is more akin to a forward or backward “pitch” along the AP axis [59,60,61,62,63,64,65,66]. Accordingly, we hypothesized that CoP’s responses to CoM changes would accentuate predominantly along the AP axis (Hypothesize 2a). Conversely, as part of our control, we anticipate that CoM will not show sensitivity to CoP changes along the AP axis. We also expected that the implication of CoP’s responses to CoM changes along the AP axis would potentially diminish its sensitivity to CoM changes along the ML axis, given the same biomechanical constraints channeling ML instability into responses along the AP axis (Hypothesis 2b). Conversely, as part of our control, we anticipate no axis-dependent discrepancy in CoM’s responses to CoP changes.*

**Hypothesis** **3.** *One confounding element in testing Hypothesis 2 is the potential for the AP leaning inclination, often known as “pitch,” to be impacted by the demands on visual attention that come with standing with one’s face front and eyes open. To account for this possibility, we varied the demands for visual tasks along the AP axis by instructing participants to complete a body-sized Trail Making Task (TMT) projected on a screen, emphasizing the “Part A” component to evaluate relatively basic cognitive processing speed. The TMT is an ideal visuomotor task to tax the postural control system along the AP axis as it engages various cognitive functions [67,68,69,70,71,72,73,74,75,76], and poor performance on the TMT has been associated with balance difficulties and an increased risk of falls [77,78,79,80,81]. We hypothesized that any accentuation in CoP’s responses to CoM changes along the AP axis caused by perturbations along the ML “roll” may be observably distinct from visual demands along the same axis. Specifically, we expected that any amplification of CoP’s responses to CoM changes along the AP axis induced by the wobble board would exceed any amplification resulting from engagement with the TMT.*

## 2. Methods

### 2.1. Participants

Forty-eight healthy young adults (M±SD age: 26±4.3 years; 24 women) participated in this study.

### 2.2. Tasks, Procedure, and Instructions to Participants

The study was conducted in the Biomechanics Research Building. A wooden wobble board measuring l×w×h=44.5×35.0×9.5cm, topped with an anti-slip pad, was utilized in the experiment. Part A of the TMT was projected onto a 1.80×1.80m screen, requiring participants to trace a path through disordered numerical sequences. Each participant underwent four distinct conditions, each consisting of two trials, resulting in eight trials per participant. To minimize potential order effects, trial sequences were randomized for each participant.

**Figure 1:**
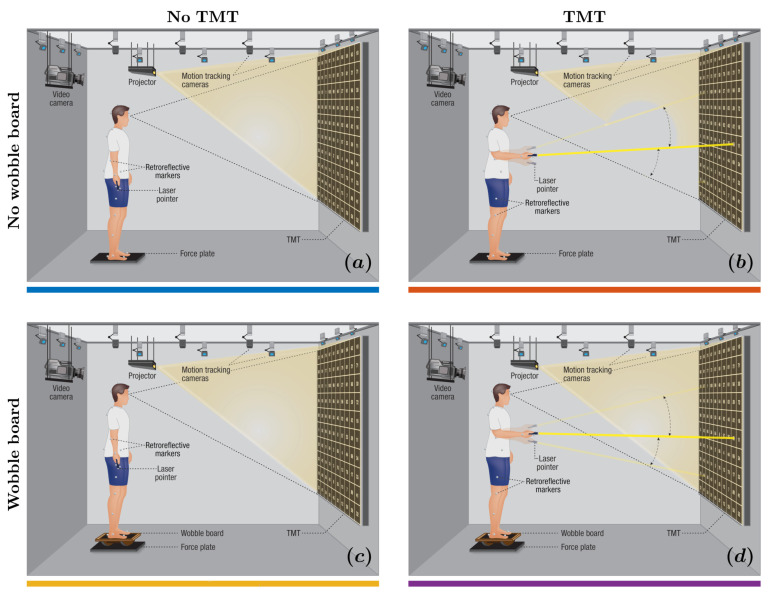
Experimental setup. We integrated a body-sized Trail Making Test (TMT) with stabilography and full-body motion tracking, requiring participants to stand either directly on a force plate or atop a wobble board inducing mediolateral instability and trace a path through randomly placed numbers projected on the screen using a laser pointer held in hand. We assessed changes in the engagement of compensatory postural adjustments (CPAs; 100–1000ms) across four conditions: (**a**) no wobble board, no TMT; (**b**) wobble board, no TMT; (**c**) wobble board, no TMT; and (**d**) wobble board, TMT.

In the “no wobble board, no TMT” condition (Figure 1a), participants maintained an upright stance on the force plate for five minutes. In the “no wobble board, TMT” condition (Figure 1b), participants maintained an upright stance on the force plate while performing the TMT using a laser pointer. In the “wobble board, no TMT” condition (Figure 1c), participants stood on an ML axis-aligned wobble board positioned centrally on the force plate, maintaining the stance for five minutes. In the “wobble board, TMT” condition (Figure 1d), participants stood on the ML axis-aligned wobble board positioned centrally on the force plate while performing the TMT using a laser pointer. During the four trials without the wobble board, participants stood barefoot at the central position of the force plate. We instructed the participants to ensure that neither foot lost contact with the force plate during each five-minute trial. Conversely, in the remaining four trials involving the wobble board, we instructed the participants that the edges of the wobble board must not make contact with the force plate. We did not provide additional instructions, such as “minimize body sway,” to avoid imposing unnatural constraints on posture, as prior research has shown that even simple instructions can alter participant attention and affect postural control strategies [82]. A distinct randomized number sequence was chosen for each TMT trial to reduce learning effects between successive trials. The TMT was shown in both the no TMT and TMT conditions to maintain consistency across all experimental scenarios. Participants underwent a familiarization session with the wobble board and received task instructions before commencing the initial trial.

We used a uniaxial (rocker-type) wobble board (44.5×35.0×9.5cm; custom-built) designed to induce mechanical instability specifically along the ML axis. The board featured a flat, nonslip top surface mounted on three fixed cylindrical supports underneath, allowing smooth and controlled rolling exclusively in the ML direction. This uniaxial design ensured consistent and repeatable lateral destabilization while minimizing the multidirectional variability and postural unpredictability often introduced by hemispherical or omnidirectional platforms.

The TMT used in this study was a body-scaled, projected version of TMT Part A, which primarily assesses visual scanning and motor speed. Participants used a handheld laser pointer to sequentially trace numbers from 1 to 100 arranged randomly in a 10×10 grid on a 1.80×1.80m screen positioned at eye level. This design emphasized visual and attentional demands in the AP axis and was expected to challenge postural control by increasing cognitive load during upright stance.

### 2.3. Stabilography

Participants stood directly or over a wobble board on a 40×60cm floor-embedded force plate (400600HPS^TM^, AMTI Inc., Watertown, MA, USA), and strain gauge transducers within the force plate registered the ground reaction forces and moments beneath participants’ feet, thus converting the mechanical deformation of strain gauges into electrical signals. The sampling rate was set to 1000Hz in accordance with the specifications of the larger study, of which this study is a part. We used these ground reaction forces and moments to compute postural CoP along the ML and AP axes.

### 2.4. Motion Tracking

Participants wore exercise or form-fitting clothing for data collection and were barefoot. Twenty-nine retroreflective markers were placed on each participant’s body utilizing the modified Helen Hayes marker set. The Helen Hayes marker set offers several benefits for motion capture, especially in biomechanics research. A primary advantage is its standardized placement, which has been extensively validated and widely used in clinical and research environments. This standardization ensures reliable, reproducible data collection across various studies and labs, enabling consistent tracking of body segments and joint movements. This marker set is also designed to cover essential anatomical landmarks while using fewer markers, reducing participant discomfort and minimizing the risk of interference during dynamic tasks. To calibrate the whole-body biomechanical model, the participants stood on the force plate with feet apart. They were instructed to hold a “T” pose, raising their arms in abduction to approximately $90^{\circ }$ and holding for 5–7 seconds. The laser pointer rested on a table in the visual field of the cameras during the calibration. We recorded marker trajectories in 3D at 100Hz using a 12-camera motion-capture system (Kestral 4200^TM^, Motion Analysis Corp., Rohnert Park, CA, USA).

### 2.5. Data Processing

#### 2.5.1. Postural Center of Mass (CoM)

In biomechanical studies involving motion capture, researchers often need to fill gaps to compensate for missing data caused by occlusions or marker loss. A common approach involves generating virtual points, especially when enough markers are available on the body segment of interest. Researchers create these virtual points based on the known configuration of markers on the segment and use them to reconstruct the trajectories of missing markers during dynamic trials. This process typically starts with a static trial, where the participant adopts a predefined pose, allowing researchers to define secondary points on the rigid body segments. These secondary points serve as references during dynamic trials, enabling the accurate reconstruction of virtual points when primary markers are obscured. In this study, researchers could not use virtual points due to insufficient marker data. As a result, they employed cubic spline interpolation to estimate the missing data, following standard practice. This method ensured a smooth and physiologically plausible reconstruction of the motion trajectory across the gap [83,84]. Combining these techniques enhanced the reliability of the kinematic analysis, particularly in determining postural CoM.

We used the Dempster model [85] to determine segment masses and spatial locations, ensuring precise assessment of body segment parameters. Simultaneously, we employed the Hanavan model [86] to compute the inertial properties of these segments. For the accurate calculation of joint centers, we adopted the Bell method [87], specifically for hip joint centers. This geometric approach utilizes the positions of two anterior superior iliac spine (ASIS) markers to define the spatial orientation of the hip joint. We defined the orientation of the pelvis by utilizing ASIS and sacral markers, thus ensuring a sturdy biomechanical model for pelvic dynamics. Likewise, we determined the locations of shoulder joints through geometric methods, leveraging the positions of right and left shoulder markers to refine our representation of upper limb mechanics. We precisely located other pivotal joints—elbows, wrists, knees, and ankles—using respective lateral and medial markers. Furthermore, we used the Terry database [88], which provided crucial iliac landmarks for accurately defining the thorax segment. All the computational analyses and definitions mentioned earlier were integrated and processed through a Visual3D^®^ (C-Motion Inc., Germantown, MD, USA) pipeline.

#### 2.5.2. Postural Center of Pressure (CoP)

We used the ground reaction forces and moments recorded by the force plate to compute postural CoP along the AP and ML axes using the formula:

$${\mathrm{CoP}}_{\mathrm{ML}}\left(t\right)=\frac{M_{x}\left(t\right)}{F_{z}\left(t\right)}+\mathrm{plate}\;{\mathrm{origin}}_{x},\;{\mathrm{CoP}}_{\mathrm{AP}}\left(t\right)=\frac{M_{y}\left(t\right)}{F_{z}\left(t\right)}+\mathrm{plate}\;{\mathrm{origin}}_{y},$$

where ${\mathrm{CoP}}_{\mathrm{ML}}\left(t\right)$ and ${\mathrm{CoP}}_{\mathrm{AP}}\left(t\right)$ are the coordinates of the CoP along the ML and AP axes at timepoint *t* and $M_{x}\left(t\right)$, and $M_{y}\left(t\right)$, $F_{z}\left(t\right)$ are directional components of the moments and forces acting on the body from the force plate.

#### 2.5.3. Signal Processing

We applied a Butterworth filter with a 60Hz cutoff frequency to the CoP trajectories along the AP and ML axes. The Butterworth filter is known for its maximally flat frequency response in the passband, which helps minimize distortions and preserve the signal’s integrity. This smooth response is particularly advantageous as it prevents the introduction of ripples that could compromise signal quality [89]. The filter was implemented using the butter() function in MATLAB 2024a (MathWorks Inc., Natick, MA, USA). We used the buttord() function to determine the minimum filter order based on specified criteria for passband and stopband ripple and attenuation. We subsequently downsampled the CoP trajectories along the AP and ML axes to 100Hz to align with the sampling rate of the CoM. For the CoM trajectories, we applied a Butterworth filter with a 6Hz cutoff frequency. This lower cutoff was chosen because the CoM represents larger-scale, slower body movements than the finer, quicker adjustments captured in CoP data. CoM movements typically fall within a lower frequency range, so a 6Hz filter preserves these important components while eliminating unwanted noise without affecting the signal’s integrity. By calculating CoP from ground reaction forces and moments and calculating CoM from marker positions before applying filters, rather than filtering these forces first, the signal’s integrity is maintained, and post-filtering minimizes distortion [90]. This approach is especially vital for studying dynamic activities, such as wobble board movements, where small CoP shifts are crucial in assessing balance and control.

### 2.6. Detrended Fluctuation Analysis (DFA)-Based Multiscale Regression Analysis

DFA computes the Hurst exponent, *H*, which quantifies the strength of long-range correlations within measurement time series [91,92]. The DFA algorithm can be succinctly summarized with two relatively simple equations:

$$F_{X}=\sqrt{\frac{\sum_{j=1}^{T-s+1}f_{X}^{2}\left(s,j\right)}{T-s}},$$

where

$$f_{X}^{2}\left(s,j\right)=\frac{\sum_{k=j}^{j+s-1}{\left(X_{k}-{\hat{X}}_{k,j}\right)}^{2}}{s-1}.$$

These equations suggest that, at various scales denoted by *s*, the *T*-length time series $X_{t}$ is segmented into non-overlapping bins of size *s*, where t=1,2,3,…,T represents the index of the time series. $X_{t}$ undergoes detrending using Ordinary Least Squares (OLS) regression within each bin. Here, $X_{t}$ denotes a real-valued time series, and $X_{k}$ represents the *k*th element obtained from the windowing process. The residuals of each scale- and window-specific regression, denoted by ${\hat{X}}_{k,j}$, are squared and then averaged. Subsequently, the square root is calculated to form the fluctuation function $F_{X}\left(s\right)$, representing the root mean squared deviation of $X_{t}$ from the local OLS regression lines. Typically, $\log F_{X}\left(s\right)$ is regressed against logs to delineate the scaling behavior inherent in $X_{t}$.

Detrended Cross-Correlation Analysis (DCCA) extends the DFA method to analyze bivariate time series concurrently [93,94,95]. In DCCA, the DFA algorithm is applied independently twice, once to each signal within a bivariate time series. This procedure yields scale-wise estimates of fluctuations (i.e., standard deviations):

$$F_{Y}=\sqrt{\frac{\sum_{j=1}^{T-s+1}f_{Y}^{2}\left(s,j\right)}{T-s}},$$

where

$$f_{Y}^{2}\left(s,j\right)=\frac{\sum_{k=j}^{j+s-1}{\left(Y_{k}-{\hat{Y}}_{k,j}\right)}^{2}}{s-1}.$$

In addition, the scale-wise covariance is estimated as:

$$F_{XY}=\sqrt{\frac{\sum_{j=1}^{T-s+1}f_{XY}^{2}\left(s,j\right)}{T-s}},$$

where

$$f_{XY}^{2}\left(s,j\right)=\frac{\sum_{k=j}^{j+s-1}\left(X_{k}-{\hat{X}}_{k,j}\right)\left(Y_{k}-{\hat{Y}}_{k,j}\right)}{s-1},$$

and the scale-wise correlation coefficient is estimated as:

$$\rho \left(s\right)=\frac{F_{XY}^{2}\left(s\right)}{F_{X}\left(s\right)\cdot F_{Y}\left(s\right)}.$$

A smooth transition from estimating correlation across multiple scales using DCCA to estimating scale-wise regression coefficients using Multiscale Regression Analysis (MRA) necessitates only a minor adjustment [57]. Unlike DCCA, a simplified version of MRA provides a symmetric estimate of the magnitude and direction of the relationship between two variables; MRA furnishes estimates of the asymmetric multiscale relationship between two variables while maintaining their original units. To transition from DCCA to MRA, an adjustment is implemented in Equation (8) by replacing the denominator with the scale-wise variance of the predictor variable:

$$\hat{\beta }\left(s\right)=\frac{F_{XY}^{2}\left(s\right)}{F_{X}^{2}\left(s\right)}.$$

where $\hat{\beta }\left(s\right)$ is the scale-wise regression coefficient obtained from regressing $Y_{t}$ on $X_{t}$. Kristoufek [57] identified the variance of $\hat{\beta }\left(s\right)$ as

$$\sigma_{\hat{\beta }\left(s\right)}^{2}=\frac{1}{T-2}\times \frac{F_{u}^{2}\left(s\right)}{F_{Y}^{2}\left(s\right)}.$$

where the scale-wise residual variance, ${\hat{F}}_{u}^{2}\left(s\right)$, is derived by applying the DFA algorithm to the scale-wise residuals, ${\hat{u}}_{t}\left(s\right)$, which are obtained as follows:

$${\hat{u}}_{t}=y_{t}-x_{t}\;\mathrm{and}\;\hat{\beta }\left(s\right)=\frac{F_{XY}^{2}\left(s\right)}{F_{X}^{2}\left(s\right)}.$$

We used MRA to analyze bivariate time series data of CoM → CoP and CoM → CoP, spanning timescales from 10 to 1000 samples (equivalent to 100–10,000ms), with intervals evenly distributed by 0.1 within the logarithmic scale.

### 2.7. Linear Mixed-Effects Modeling of $\hat{\beta }$

We first calculated the average $\hat{\beta }$ across all timescales associated with CPA1, CPA2, and CPA3. We submitted these values to linear mixed-effects modeling. We used two separate linear mixed-effects models to (i) evaluate the overall CoP-to-CoM and CoM-to-CoP effects and (ii) evaluate CoP-to-CoM and CoM-to-CoP effects specifically along the ML and AP axes. The model included the fixed effects of trial (“T2” for trial 2 as opposed to trial 1), support condition (“WB” for wobble board as opposed to no wobble board), task condition (“TMT” as opposed to no TMT), CPA (“CPA2” and “CPA2” as opposed to CPA3), and type (“Original” as opposed to the corresponding IAAFT surrogates). The second model also included the fixed effect of the axis (“ML” as opposed to AP). We included the random factor of participant identity by allowing the intercept to vary across participants. We performed all statistical analyses in R [96] using the function lmer() from the package “lme4” [97]. We set the threshold for statistical significance at the alpha level of 0.05 using the package “lmerTest” [98]. The linear mixed-effect models yielded coefficients for each covariate, representing the average change in $\hat{\beta }$. Corresponding to each coefficient was a standard error (SE), denoting the variability around the mean change in $\hat{\beta }$. In our subsequent analysis, we present the estimated coefficients from the linear mixed-effect model in the format B±SE, alongside the associated *t*-statistic, calculated as B/SE, and the corresponding *p*-value.

We also conducted an exploratory analysis in which sex (male vs. female) was included as an additional fixed effect to examine potential sex-based differences in CoP–CoM dynamics. This analysis yielded no significant main effects of sex or interactions between sex and any task variables across timescales or conditions. To maintain model parsimony and avoid overfitting, we excluded sex from the final reported models.

## 3. Results

### 3.1. Depicting the Wobble-Board Induced Accentuation of ML Sway

**Figure 2:**
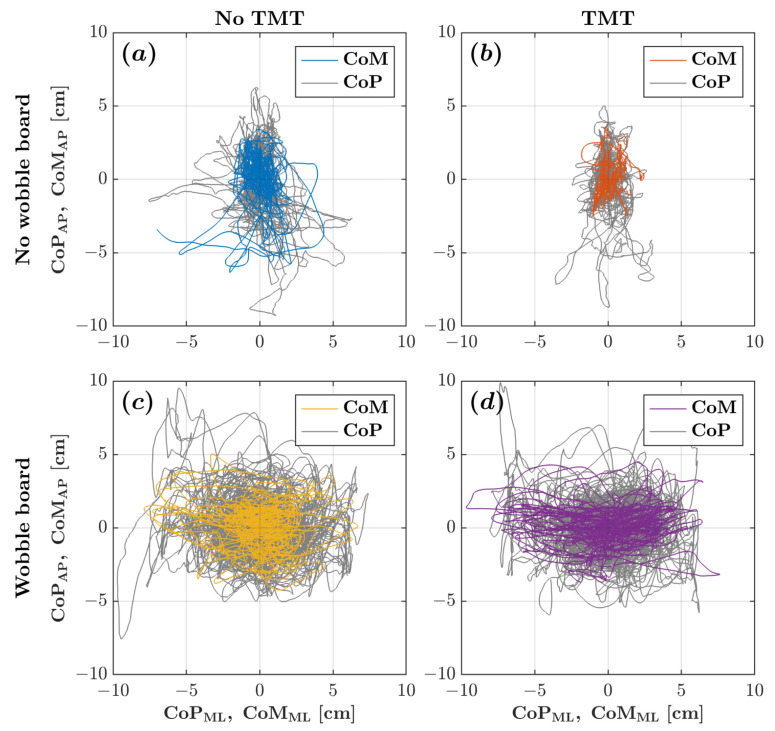
CoM (colored traces) and CoP (gray traces) planar trajectories for a representative participant across the four experimental conditions: (**a**) no wobble board, no TMT; (**b**) no wobble board, TMT; (**c**) wobble board, no TMT; and (**d**) wobble board, TMT.

**Figure 3:**
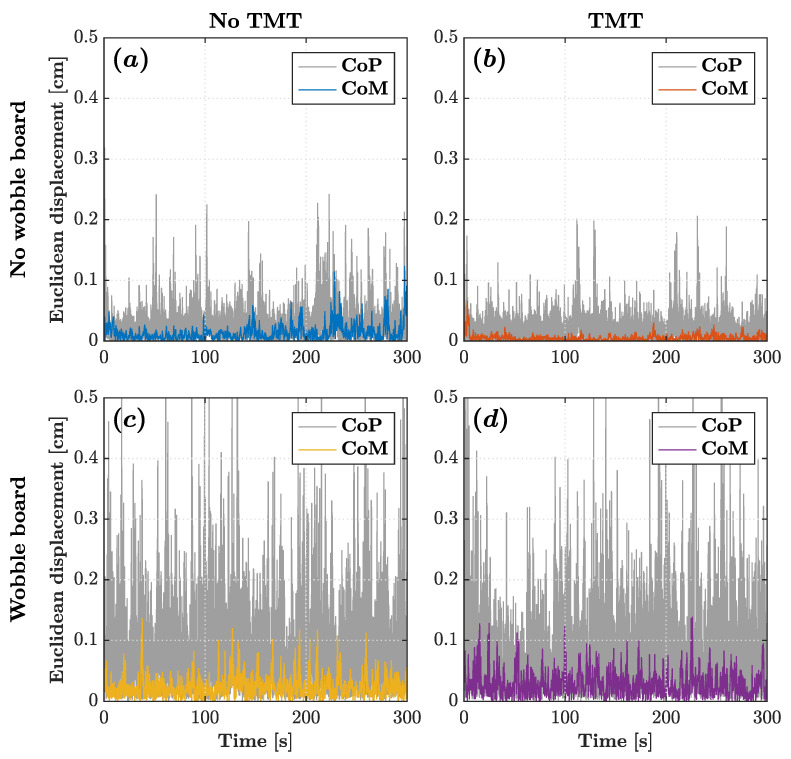
CoM (colored traces) and CoP (gray traces) planar displacement time series for a representative participant across the four experimental conditions: (**a**) no wobble board, no TMT; (**b**) no wobble board, TMT; (**c**) wobble board, no TMT; and (**d**) wobble board, TMT.

Without the wobble board, the CoM and CoP planar trajectories exhibited an elongated path along the AP axis, reflecting the conventional understanding that posture tends to be less stable along the AP axis. However, as depicted in Figure 2, when the wobble board was introduced, the CoP trajectory notably expanded its dispersion along the ML axis, showcasing a considerably broader range of postural sway along the ML axis compared to the AP axis. Figure 3 depicts the corresponding CoM and CoP planar displacements for the four experimental conditions.

### 3.2. Hypothesis 1: Wobble Board Attenuates CoP’s Responses to CoM Changes

**Figure 4:**
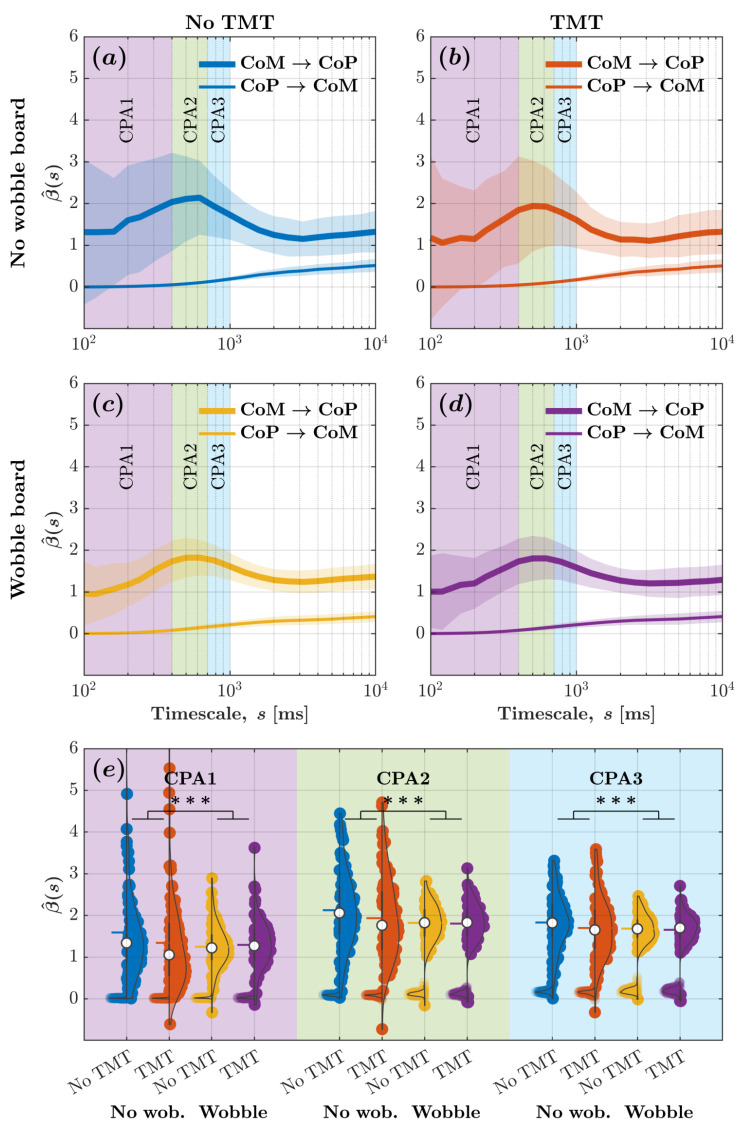
Wobble board attenuated CoM-to-CoP effects indicative of a reduction in CoM-mediated compensatory postural adjustments, as evinced by diminution of the peak in $\hat{\beta }$ values for CoM and CoP planar displacements. (**a**–**d**) The metric $\hat{\beta }$ encoding CoM-to-CoP and CoP-to-CoM effects vs. log-timescale, $\hat{\beta }$ vs. logs, the relationship across distinct timescales corresponding to the compensatory postural responses (CPA1: 100–400ms; CPA2: 400–700ms; CPA3: 700–1000ms) for the four experimental conditions: (**a**) no wobble board, no TMT; (**b**) no wobble board, TMT; (**c**) wobble board, no TMT; and (**d**) wobble board, TMT. Solid lines indicate *mean* and shaded regions indicate ±1SD (N=96;48participants×2trials/participant). (**e**) *Mean* $\hat{\beta }$ values encoding CoM-to-CoP and CoP-to-CoM effects across all timescales corresponding to CPA1, CPA2, and CPA3 for the four experimental conditions. Each violin plot’s left and right half depicts $\hat{\beta }$ values encoding CoM-to-CoP and CoP-to-CoM effects, respectively. Horizontal bars indicate *mean*, and white circles indicate *median* (N=96;48participants×2trials/participant). *** p<0.001.

Figure 4 depicts the $\hat{\beta }$-vs.-log-timescale relationship quantifying the bidirectional relationship between CoM and CoP planar displacements across timescales corresponding to the CPA1 (100–400ms), CPA2 (400–700ms), CPA3 (700–1000ms). These curves indicate that while the CoP responded to changes in the CoM, the CoM did not significantly respond to changes in the CoP. It also indicates that the wobble board attenuated CoM-to-CoP effects, that is, CoP’s responses to CoM changes, especially within timescales spanning 100–400ms (Figure 4c,d as opposed to Figure 4a,b).

For evaluating how task constraints influence CoM–CoP coupling, we calculated the average $\hat{\beta }$ for CoM and CoP planar displacement across all timescales associated with CPA1, CPA2, and CPA3. We submitted these $\hat{\beta }$ values to linear mixed-effects modeling. Regression coefficients validated the aforementioned trends (Figure 4e; Table 1). The introduction of the wobble board accentuated CoP-to-CoM effects by $1.326\times 10^{-2}\pm 3.491\times 10^{-3}$ $\left(t=3.799,\;p=1.490\times 10^{-4}\right)$, but the engagement with the TMT did not influence CoP-to-CoM effects (allp>0.05). The accentuation of CoP-to-CoM effects due to the wobble board was greater for CPA2 than CPA1 by $6.269\times 10^{-2}\pm 4.937\times 10^{-3}$ $\left(t=12.697,\;p<2.000\times 10^{-16}\right)$ and for CPA3 than CPA1 by $8.494\times 10^{-2}\pm 4.937\times 10^{-3}$ $\left(t=17.205,\;p<2.000\times 10^{-16}\right)$. CoP-to-CoM effects did show a marginal increase in $\hat{\beta }$ values for CPA2 than CPA1 by $6.634\times 10^{-2}\pm 4.275\times 10^{-3}$ $\left(t=15.517,\;p<2.000\times 10^{-16}\right)$ and for CPA3 than CPA1 by $1.413\times 10^{-1}\pm 4.275\times 10^{-3}$ $\left(t=33.047,\;p<2.000\times 10^{-16}\right)$. CoM-to-CoP effects with $\hat{\beta }$ values of $1.812\times 10^{0}\pm 4.275\times 10^{-3}$ exceeded CoP-to-CoM effects with $\hat{\beta }$ values of $3.189\times 10^{-2}\pm 3.023\times 10^{-3}$ $\left(t=423.793,\;p<2.000\times 10^{-16}\right)$. The introduction of the wobble board attenuated CoM-to-CoP effects by $1.158\times 10^{0}\pm 4.937\times 10^{-3}$ compared to standing directly on the force plate $\left(t=-234.618,\;p<2.000\times 10^{-16}\right)$, and the engagement with the TMT attenuated CoM-to-CoP effects by $9.096\times 10^{-2}\pm 4.937\times 10^{-2}$ compared to simply standing upright directly on the force plate $\left(t=18.425,\;p<2.000\times 10^{-16}\right)$. Compared to CPA1, CoM-to-CoP effects were accentuated by $1.138\times 10^{0}\pm 6.046\times 10^{-3}$ for CPA2 $\left(t=188.228,\;p<2.000\times 10^{-16}\right)$ and by $8.611\times 10^{-1}\pm 6.046\times 10^{-3}$ for CPA3 $\left(t=142.409,\;p<2.000\times 10^{-16}\right)$. Compared to CPA1, the attenuation of CoM-to-CoP effects due to the wobble board was smaller for CPA2 $(B\pm SE=-5.851\times 10^{-1}\pm 6.982\times 10^{-3}$, $t=-83.799,\;p<2.000\times 10^{-16})$ and CPA3 $(B\pm SE=-3.858\times 10^{-1}\pm 6.982\times 10^{-3},$ $t=-55.260,\;p<2.000\times 10^{-16})$. Likewise, compared to CPA1, the accentuation of CoM-to-CoP effects due to the TMT was marginally more for CPA2 $\left(B\pm SE=1.975\times 10^{-1}\pm 6.982\times 10^{-3},t=28.291,\;p<2.000\times 10^{-16}\right)$ and CPA3 $\left(B\pm SE=2.307\times 10^{-1}\pm 6.982\times 10^{-3},t=33.040,\;p<2.000\times 10^{-16}\right)$. These results contradicted Hypothesis 1; instead of accentuating CoP’s overall (planar) responses to CoM changes, we found that the wobble board attenuated CoP’s responses to CoM changes, especially within timescales spanning 100–400ms.

### 3.3. Hypothesis 2: Wobble Board Accentuates CoP’s Responses to CoM Changes Along the AP Axis and Attenuates Along the ML Axis

**Figure 5:**
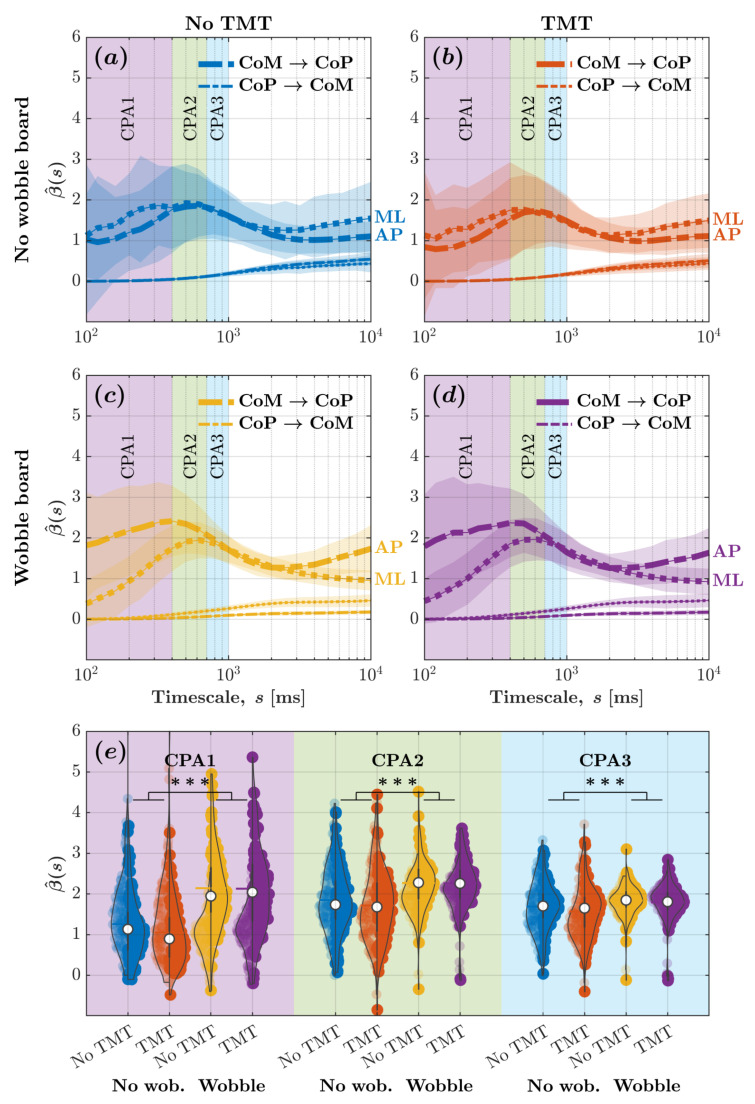
Despite rendering posture more unstable along the ML axis, the wobble board accentuated the CoM-to-CoP effect significantly more along the AP axis and attenuated the CoM-to-CoP effect significantly more along the ML axis. (**a**–**d**) The metric $\hat{\beta }$ encoding CoP-to-CoM and CoM-to-CoP effects along the ML and AP axes vs. log-timescale, $\hat{\beta }$ vs. logs, the relationship across distinct timescales corresponding to the compensatory postural responses (CPA1: 100–400ms; CPA2: 400–700ms; CPA3: 700–1000ms) for the four experimental conditions: (**a**) no wobble board, no TMT; (**b**) no wobble board, TMT; (**c**) wobble board, no TMT; and (**d**) wobble board, TMT. Solid lines indicate *mean* and shaded regions indicate ±1SD (N=96;48participants×2trials/participant). (**e**) *Mean* $\hat{\beta }$ values encoding CoM-to-CoP and CoP-to-CoM effects along the ML and AP axes across all timescales corresponding to CPA1, CPA2, and CPA3 for the four experimental conditions. Each violin plot’s left and right half depicts $\hat{\beta }$ values encoding CoM-to-CoP effects along the ML and AP axes, respectively. Horizontal bars indicate *mean*, and white circles indicate *median* (N=96;48participants×2trials/participant). *** p<0.001.

Figure 5 depicts the $\hat{\beta }$-vs.-log-timescale relationship quantifying the bidirectional relationship between CoM and CoP displacements along the ML and AP axis across timescales corresponding to the CPA1 (100–400ms), CPA2 (400–700ms), CPA3 (700–1000ms). These curves add nuance to those in Figure 4, indicating that while the CoP responded to changes in the CoM along both the ML and AP axes, the CoM did not significantly respond to changes in the CoP along either axes. It also indicates that the wobble board attenuated CoM-to-CoP effects, that is, CoP’s responses to CoM changes, along the ML axis and accentuated CoM-to-CoP effects along the AP axis, especially within timescales spanning 100–400ms (Figure 5c,d as opposed to Figure 5a,b).

For evaluating how task constraints influence CoM–CoP coupling, we calculated the average $\hat{\beta }$ for CoM and CoP displacements along the ML and AP axes across all timescales associated with CPA1, CPA2, and CPA3. We submitted these $\hat{\beta }$ values to linear mixed-effects modeling. Regression coefficients validated the aforementioned trends (Figure 5e; Table 2). Neither the wobble board’s introduction nor the TMT engagement influenced CoP-to-CoM effects along the ML axis (allp>0.05). CoM-to-CoP effects along the ML axis, with $\hat{\beta }$ values of $1.812\times 10^{0}\pm 4.657\times 10^{-2}$, exceeded CoP-to-CoM effects along the ML axis, which had $\hat{\beta }$ values of $3.189\times 10^{-2}\pm 3.587\times 10^{-2}$ (t=0.889, p=0.374). The introduction of the wobble board attenuated CoM-to-CoP effects along the ML axis by $1.158\times 10^{0}\pm 5.337\times 10^{-2}$ compared to standing directly on the force plate $\left(t=-21.550,\;p<2.000\times 10^{-16}\right)$, but the TMT did not influence CoM-to-CoP effects (p>0.05). The $\hat{\beta }$ values for CoM-to-CoP effects were smaller along the AP axis compared to the ML axis by $3.116\times 10^{-1}\pm 6.586\times 10^{-2}$ $\left(t=-4.732,\;p=2.290\times 10^{-6}\right)$. This effect was reversed by the wobble board $(B\pm SE=9.511\times 10^{-1}\pm 7.605\times 10^{-2},$ $t=12.507,\;p<2.000\times 10^{-16})$ and remained unaffected by the engagement with the TMT $\hat{\beta }$ values (p>0.05). These results contradicted Hypothesis 2; the wobble board accentuated CoP’s responses to CoM changes along the AP axis and attenuated along the ML axis, especially within timescales spanning 100–400ms.

Compared to CPA1, CoM-to-CoP effects along the ML axis were accentuated by $1.138\times 10^{0}\pm 6.586\times 10^{-2}$ for CPA2 $\left(t=17.281,\;p<2.000\times 10^{-16}\right)$ and by $8.611\times 10^{-1}\pm 6.586\times 10^{-2}$ for CPA3 $\left(t=13.074,\;p<2.000\times 10^{-16}\right)$. This accentuation disappeared along the AP axis (CPA2 vs. CPA1: $B\pm SE=-6.442\times 10^{-1}\pm 9.314\times 10^{-2},t=-6.916,$ $p=5.270\times 10^{-12}$; CPA3 vs. CPA1: $B\pm SE=-7.195\times 10^{-1}\pm 9.314\times 10^{-2},$ $t=-7.725,\;p=1.370\times 10^{-14})$. The wobble board reversed the differences in CoM-to-CoP effects along the ML axis between CPA1 and CPA2 $(-5.851\times 10^{-1}\pm 7.605\times 10^{-2},$ $t=-7.694,\;p=1.740\times 10^{-14})$ as well as between CPA1 and CPA3 $\left(-3.858\times 10^{-1}\pm 7.605\times 10^{-2},t=-5.073,\;p=4.060\times 10^{-7}\right)$. This wobble-board-induced reversal was not observed along the AP axis (CPA2 vs. CPA1: $B\pm SE=5.337\times 10^{-1}\pm 1.075\times 10^{-1},$ $t=4.963,\;p=7.200\times 10^{-7}$; CPA3 vs. CPA1: $B\pm SE=4.617\times 10^{-1}\pm 1.075\times 10^{-1},$ $t=4.293,\;p=1.800\times 10^{-5})$. In contrast, the TMT marginally accentuated the difference in $\hat{\beta }$ values between CPA1 and CPA2 $(B\pm SE=1.975\times 10^{-1}\pm 7.605\times 10^{-3},t=2.597,$ $p=9.420\times 10^{-3})$ and between CPA1 and CPA3 $(B\pm SE=2.307\times 10^{-1}\pm 7.605\times 10^{-2},$ $t=3.033,\;p=2.430\times 10^{-3})$. The engagement with the TMT did not affect these trends (p>0.05). These results confirmed that the observed effects of the wobble board on CoP responses to CoM changes for CPA1 did not generalize to CPA2 and CPA3, establishing the time-specificity of the changes in the coordination and compensatory activity of bodily DoFs due to the instability induced by the wobble board.

### 3.4. Hypothesis 3: The Wobble-Board Induced Changes in CoP’s Responses to CoM Changes Exceed Any Effects of TMT

Supporting Hypothesis 3, all observed changes in CoM-to-CoP effects due to the wobble board were significantly larger than any changes caused by the TMT, prompting visual attention along the AP axis. This was true for both CoM and CoP planar displacements (see Figure 4b,d as opposed to Figure 4a,c; see also Figure 4e) and for CoM and CoP displacements along either the AP or ML axes (Figure 5b,d as opposed to Figure 5a,c; see also Figure 5e). Regression coefficients confirmed these trends (Table 1 and Table 2).

### 3.5. TMT Had No Significant Effect on CoM–CoP Dynamics

Across all analyses, the TMT had no statistically significant impact on the multiscale structure of CoM–CoP dynamics. Linear mixed-effects models revealed no reliable main effects of the TMT condition on CoP-to-CoM or CoM-to-CoP effects, whether evaluated across the full planar displacement or along the individual ML and AP axes. This null result held across all examined timescale ranges (CPA1–CPA3), as shown in Figure 4 and Figure 5, where curves for TMT and no-TMT conditions largely overlapped. While some interaction terms involving TMT and timescale reached statistical significance in the model (e.g., TMT × CPA2, TMT × CPA3), these effects were modest and did not substantively alter the overall directionality or magnitude of CoM–CoP coupling. These findings suggest that the visuomotor demands imposed by TMT Part A were insufficient to meaningfully perturb multiscale postural control in healthy young adults.

## 4. Discussion

### 4.1. Key Findings

This study investigated the complex interplay of bodily degrees of freedom (DoFs) in governing the collective variable of CoM–CoP dynamics during upright stance, using a multiscale analytical framework. Participants performed a combination of experimental tasks that introduced mechanical instability primarily along the mediolateral (ML) axis using a wobble board, and imposed visuomotor demands primarily along the anteroposterior (AP) axis through a body-scaled version of the Trail Making Task (TMT). Using Multiscale Regression Analysis (MRA), rooted in Detrended Fluctuation Analysis (DFA), we examined CoP-to-CoM and CoM-to-CoP interactions across timescales ranging from 100ms to 10s.

We formulated three hypotheses. Hypothesis 1 predicted that wobble board-induced instability would enhance CoP’s responses to CoM movements, especially within the 100–400ms window, reflecting increased compensatory activity. Hypothesis 2 proposed direction-specific effects: an increase in CoM-to-CoP responses along the AP axis and a decrease along the ML axis. Hypothesis 3 anticipated that any effects induced by the wobble board would exceed those attributable to the TMT. Our results revealed a more nuanced picture. Contrary to Hypothesis 1, wobble board instability attenuated overall CoM-to-CoP effects within the targeted timescales. However, consistent with Hypothesis 2, this attenuation was directionally specific—CoM-to-CoP effects increased along the AP axis and decreased along the ML axis. Hypothesis 3 was also supported, as the TMT produced no statistically meaningful changes in CoM–CoP dynamics. Notably, CoM showed minimal sensitivity to CoP across all conditions. These findings highlight the timescale- and direction-specific nature of postural adaptations and underscore the importance of biomechanical constraints in shaping compensatory strategies. They further support the potential of mechanically destabilizing interventions, such as wobble boards, for eliciting adaptive postural control mechanisms relevant to clinical populations at risk for falls.

### 4.2. Interpretation of Wobble Board Effects

The main finding of this study emphasizes the impact of wobble board instability on accentuating the CoP’s responses to CoM movements. While we did not observe a notable increase in CoM and CoP planar displacements (i.e., along both the AP and ML axes), we detected heightened CoP responses to CoM movements along the AP axis and reduced CoP responses along the ML axis. This finding represents a more nuanced expansion of previous research, highlighting the distinct specificity of manipulated instability (spanning 300s) on heightened coordination and compensatory activity of bodily DoFs (spanning 100–400ms), indicating a highly specific, finely-tuned postural responses to long-scale task manipulation. How do we explain this orthogonal discrepancy and ML instability resulting in CoP responses along the AP axis? Human balance is inherently a 3D challenge, where disturbances in one direction often affect stability in others [59,60,61,62,63,64,65,66]. For example, when balance is disrupted laterally, the body compensates by engaging muscles and adjusting posture in both the side-to-side and front-to-back directions to maintain stability. While lateral (ML) instability naturally increases postural responses along the ML axis, it also heightens responses along the AP axis while reducing them along the ML axis. This may result from asymmetries in the biomechanical constraints of postural control, where certain muscle groups are better suited to generating corrective forces in specific directions. For instance, anterior muscles are more effective at moving the body forward to counteract lateral sway. We propose that the orthogonality of these coordinative and compensatory responses to ML instability induced by the wobble board is closely linked to the CoM. The primary goal of postural control is to keep the CoM within the stable boundaries of the BoS [1,2,3]. In standing posture, the BoS is typically longer along the AP axis (foot length) than the ML axis (foot width). A lateral perturbation brings the CoM closer to the BoS edge, so the body may shift the CoM forward or backward within the longer AP axis to regain a more stable position.

Although our title highlights the enhancement of CoP’s responses to CoM movement, the results reveal a directionally specific modulation rather than a uniform increase. We observed an amplification of CoM-to-CoP effects along the AP axis, accompanied by a reduction along the ML axis. This pattern suggests not a contradiction but a redistribution of compensatory mechanisms tailored to the nature of the imposed instability. Given the longer base of support and greater biomechanical leverage along the AP axis, the postural control system may preferentially engage AP-directed strategies when challenged in the ML direction. Such a shift likely reflects an adaptive reorganization of available degrees of freedom to preserve overall stability in the face of directionally asymmetric task demands.

One of the most significant findings is the attenuation of CoP-to-CoM effects along the ML axis. How can we interpret this result? The notably increased lateral displacement of the CoM with the wobble board suggests a potential increase in body sway. When this happens, the primary challenge for the supporting limbs is to counteract the gravitational load acting on the trunk and swinging leg at the hip joint. In healthy individuals, this gravitational moment is counteracted by an active abduction hip moment and a passive acceleration moment [99,100]. The greater lateral displacement of the CoM implies that participants may struggle to generate sufficient active hip abductor/adductor torque to prevent the pelvis and trunk from dropping toward the side of the swing leg. Instead, they may rely on passive lateral acceleration momentum, requiring less muscular effort to limit CoM excursions along the ML axis. Additionally, participants may attempt to arrest the forward progression of the CoM by converting forward momentum into lateral momentum. Both of these control strategies not only contribute to increased lateral CoM displacement but also diminish CoP-to-CoM effects along the ML axis. The increased influence of the CoM on the CoP in AP is significant, given that most daily activities, such as walking or reaching, rely heavily on stability in this direction. Research indicates that the risk of falls is generally greater along the AP axis than the ML axis, primarily because forward and backward movements, common in daily activities, require precise postural control. For example, older adults and stroke survivors, who are at higher risk for falls, show a greater tendency for instability along the AP direction, which has been linked to a higher incidence of falls along this axis [101,102]. The observed increase in CoM–CoP interaction along the AP axis indicates that wobble board training enhances dynamic stability, which is crucial for preventing falls, particularly in the forward or backward directions. The study’s results align with previous research demonstrating that CoP metrics, especially along the AP direction, are key postural control and fall risk indicators. Thus, incorporating wobble board exercises into balance training programs could be a valuable strategy to improve stability along the AP axis, thereby reducing the likelihood of falls, particularly in populations at higher risk [103,104,105].

### 4.3. Interpretation of TMT Effects

Finally, we found that the pronounced impact of ML instability induced by the wobble board on CoM-to-CoP effects along the AP axis surpassed the nonsignificant impact of TMTs. As discussed above, TMTs engage various cognitive functions [67,68,69,70,71,72,73,74,75,76] and poor performance in TMT is associated with poorer balance and an elevated risk of falls [77,78,79,80,81]. Contrary to the expectation arising from these findings, we observed no TMT-related changes in CoM–CoP dynamics. Even simple visual tasks, such as fixating on a support surface, suffice to change the multiscale structure of CoP [106,107]. In our study, the TMT did not significantly alter CoP–CoM dynamics. The lack of a significant TMT effect may reflect the limited cognitive load imposed by TMT Part A, which primarily assesses visual scanning and simple processing speed. More demanding variants, such as TMT Part B or dual-task paradigms involving executive functions like task switching or response inhibition, may be necessary to elicit meaningful changes in postural coordination. Additionally, participants in our study were healthy young adults likely to possess substantial cognitive reserve, which may have minimized the observable impact of the visuomotor task on balance control.

### 4.4. Theoretical Implications

We aim to elucidate the implications of our findings for theoretical frameworks concerning postural control. Models of postural control, which often incorporate closed-loop responses to perturbations, typically rely on sensory feedback about an internal model. Within this framework, control entails minimizing the disparity between afferent feedback and a representation of the current bodily position—for example, of CoM or some specific configuration of the body, augmented by “efference copies” indicating intended positional adjustments [108,109,110]. An implicit assumption of such models is that the nervous system must actively adjust control parameters to minimize this discrepancy by operating on the so-called “error” [111,112,113]. We anticipate that the CoP’s responses to CoM changes are not explicitly prescribed but emerge due to two factors. Firstly, the plausibility of representation necessitates ergodicity, that is, any segment of the CoM and CoP trajectories encapsulates the entire trajectories. However, ergodicity is routinely disrupted by the multiplicative, multiscale structure [114,115,116,117,118,119], and postural control is no exception [115]. Secondly, the multiplicative, multiscale, ergodicity-breaking structure of movement variability facilitates the adaptive utilization of sensory information not only in posture [106,107,120,121,122,123,124,125,126,127,128,129,130,131,132,133,134,135] but also in perception-action cycles encompassing various suprapostural tasks with diverse cognitive demands [136,137,138,139,140,141,142,143]. Consequently, the observed CoM-to-CoP effects cannot be ascribed to a linear feedback-based model but rather suggest an emergent collective variable that arises as individuals navigate diverse organismal, task-oriented, and environmental constraints.

The immediate question arises: How do the CoM–CoP dynamics emerge as the collective variable governing postural control? As evident from the aforementioned references, exploring the multiplicative, multiscale structure of postural control has been deeply rooted in a longstanding tradition. This endeavor has significantly advanced our understanding, moving us beyond overly simplistic linear models towards nonlinear frameworks that acknowledge human physiology’s complexity, hierarchy, and multiplicative nature [30]. However, these efforts have often refrained from the specifics of sensorimotor neurophysiology. This challenge has been further compounded by the difficulty in identifying bidirectional relationships and their dependence across measurement scales. As an attempt to reconcile these multiscale approaches to postural control with sensorimotor neurophysiology, we previously identified non-Gaussian patterns in CoP displacement specific to long-latency reflexes, short-latency reflexes, and compensatory postural adjustments vary with destabilizing postural perturbations due to wobble board [144]. This study complements this endeavor by pinpointing the timescale of 300–400ms exhibiting the most pronounced CoP’s responses to CoM changes. If the decline in postural control is attributed to the sluggishness of various sensorimotor processes preceding the CoM–CoP dynamics due to aging or neurodegenerative disorders cf. [50,145,146,147,148], we would expect to observe a delayed peak in CoM-to-CoP effects. Conversely, alterations in the magnitude of the peak of CoM-to-CoP effects may indicate varying sensitivities to task constraints [55].

### 4.5. Clinical Implications and Limitations

While our findings provide key insights into the scale-specific CoP–CoM dynamics in healthy young adults, caution is warranted in generalizing these results to older adults or individuals with clinical balance impairments. These populations often exhibit distinct sensorimotor reflex profiles and altered strategies due to age-related degeneration, neurological conditions, or musculoskeletal deficits. Future research should extend this paradigm to assess how pathological changes influence the multiscale coordination and compensatory mechanisms underlying postural control.

Although the general benefits of wobble board training for balance are well documented, our study adds mechanistic clarity by showing how direction- and timescale-specific CoP–CoM dynamics are reorganized under controlled biomechanical instability. This multiscale perspective offers a novel explanatory framework for interpreting the efficacy of wobble board interventions and may inform more targeted clinical strategies for populations with impaired postural control. Wobble boards have been shown to improve balance in the elderly [149,150] and post-stroke individuals [151,152], boost muscle response times and perceived stability in those with unstable ankles [153,154], alleviate symptoms of stage-2 ankle sprains [155], and enhance proprioception in people with functional ankle instability [156,157]. In athletic contexts, wobble board training refines counter-rotation mechanisms essential for stability on unstable surfaces [158] and reduces postural sway in both the mediolateral and anteroposterior directions [159,160,161,162]. These results advance previous research in two key ways. First, they shed light on how wobble boards enhance balance, highlighting their ability to stimulate long-latency reflexes, as shown in our earlier study [144], of which this study is an extension. Second, we found that wobble boards amplify the body’s ability to coordinate and compensate for shifting CoM by adjusting the CoP. For example, when standing on a wobble board, the body must constantly adjust CoP to prevent falling, strengthening these compensatory mechanisms. Since CoM reflects the entire body’s response to destabilizing forces, wobble board interventions may be more effective than exercises targeting only specific body regions. Moreover, the neuroplastic effects of wobble board training could lead to long-term improvements in balance. Research has shown that balance training fosters corticospinal reorganization, meaning the brain and spinal cord adapt to enhance balance control [163,164,165,166,167,168,169,170,171,172]. This study reinforces prior work suggesting that CoM-to-CoP dynamics act as a collective variable for maintaining CoM stability within the base of support [6,7]. These dynamics support the use of real-time biofeedback and position wobble board training as a low-cost strategy to improve balance in individuals with postural deficits.

More specifically, the current findings offer a foundation for individualized, axis-specific rehabilitation protocols based on how mechanical instability reshapes compensatory postural strategies across timescales and directions. For instance, interventions that amplify AP-directed CoP responses in response to ML challenges may be especially beneficial for older adults, stroke survivors, or those with Parkinsonian gait—populations known to struggle with lateral instability and impaired anticipatory postural adjustments. Incorporating wobble boards into clinical assessments could also serve as a scalable and practical diagnostic implements for detecting subtle coordination deficits not observable under static balance tasks.

## 5. Conclusions

This study investigated how mechanical instability induced by a wobble board reorganizes the multiscale relationship between CoM and CoP during upright stance. While overall CoP responses to CoM movements were attenuated, we observed a direction-specific enhancement along the AP axis and a reduction along the ML axis. These findings reveal a redistribution of compensatory postural strategies under biomechanical constraint, emphasizing the role of direction-sensitive adaptations in maintaining balance. By identifying the timescales and axes at which compensatory mechanisms are recruited, this work offers mechanistic insight into CoP–CoM coordination and highlights the utility of multiscale methods for studying postural control. The results support the clinical potential of wobble board training as a targeted intervention for enhancing dynamic stability and mitigating fall risk, particularly in populations with impaired balance.

## Figures and Tables

**Table 1:** Outcomes of the linear mixed-effects model ^1^ examining the influence of trial (“T2” for trial 2 as opposed to trial 1), support condition (“WB” for wobble board as opposed to no wobble board), task condition (“TMT” as opposed to no TMT), the mechanism (“CPA2” and “CPA2” as opposed to CPA3), and CoM–CoP directionality (CoM → CoP as opposed to CoP → CoM) on $\hat{\beta }$ coefficient quantifying the relationship between CoM and CoP planar displacements.

Factor	B±SE	*t*	*p* ^2^
(Intercept)	$3.189\times 10^{-2}\pm 3.106\times 10^{-3}$	10.265	$<2\times 10^{-16}$
trialT2	$9.474\times 10^{-15}\pm 1.425\times 10^{-3}$	0.000	1.000
supportConditionWB	$1.326\times 10^{-2}\pm 3.491\times 10^{-3}$	3.799	$1.490\times 10^{-4}$
taskConditionTMT	$-6.592\times 10^{-3}\pm 3.491\times 10^{-3}$	−1.888	0.059
mechanismCPA2	$6.634\times 10^{-2}\pm 4.275\times 10^{-3}$	15.517	$<2\times 10^{-16}$
mechanismCPA3	$1.413\times 10^{-1}\pm 4.275\times 10^{-3}$	33.047	$<2\times 10^{-16}$
CoM → CoP	$1.812\times 10^{0}\pm 4.275\times 10^{-3}$	423.793	$<2\times 10^{-16}$
supportConditionWB:mechanismCPA2	$6.269\times 10^{-2}\pm 4.937\times 10^{-3}$	12.697	$<2\times 10^{-16}$
supportConditionWB:mechanismCPA3	$8.494\times 10^{-2}\pm 4.937\times 10^{-3}$	17.205	$<2\times 10^{-16}$
taskConditionTMT:mechanismCPA2	$-1.183\times 10^{-4}\pm 4.937\times 10^{-3}$	−0.024	0.981
taskConditionTMT:mechanismCPA3	$-6.512\times 10^{-3}\pm 4.937\times 10^{-3}$	−1.319	0.187
supportConditionWB:CoM → CoP	$-1.158\times 10^{0}\pm 4.937\times 10^{-3}$	−234.618	$<2\times 10^{-16}$
taskConditionTMT:CoM → CoP	$-9.096\times 10^{-2}\pm 4.937\times 10^{-3}$	−18.425	$<2\times 10^{-16}$
mechanismCPA2:CoM → CoP	$1.138\times 10^{0}\pm 6.046\times 10^{-3}$	188.228	$<2\times 10^{-16}$
mechanismCPA3:CoM → CoP	$8.611\times 10^{-1}\pm 6.046\times 10^{-3}$	142.409	$<2\times 10^{-16}$
supportConditionWB:mechanismCPA2:CoM → CoP	$-5.851\times 10^{-1}\pm 6.982\times 10^{-3}$	−83.799	$<2\times 10^{-16}$
supportConditionWB:mechanismCPA3:CoM → CoP	$-3.858\times 10^{-1}\pm 6.982\times 10^{-3}$	−55.260	$<2\times 10^{-16}$
taskConditionTMT:mechanismCPA2:CoM → CoP	$1.975\times 10^{-1}\pm 6.982\times 10^{-3}$	28.291	$<2\times 10^{-16}$
taskConditionTMT:mechanismCPA3:CoM → CoP	$2.307\times 10^{-1}\pm 6.982\times 10^{-3}$	33.040	$<2\times 10^{-16}$

[1] $\hat{\beta }∼\mathrm{trial}+\left(\mathrm{supportCondition}+\mathrm{taskCondition}\right)*\mathrm{mechanism}*\mathrm{directionality}+\left(1|\mathrm{participant}\right)$. [2] Boldfaced values indicate statistical significance at p<0.05.

**Table 2:** Outcomes of the linear mixed-effects model ^1^ examining the influence of trial (“T2” for trial 2 as opposed to trial 1), support condition (“WB” for wobble board as opposed to no wobble board), task condition (“TMT” as opposed to no TMT), the mechanism (“CPA2” and “CPA3” as opposed to CPA1), CoM–CoP directionality (CoM → CoP as opposed to CoP → CoM), and axis (“AP” as opposed to ML) on $\hat{\beta }$ coefficient quantifying the relationship between CoM and CoP displacements along the ML and AP axes.

Factor	B±SE	*t*	*p* ^2^
(Intercept)	$3.189\times 10^{-2}\pm 3.587\times 10^{-2}$	0.889	0.374
trialT2	$-5.526\times 10^{-3}\pm 1.098\times 10^{-2}$	−0.503	0.615
supportConditionWB	$1.326\times 10^{-2}\pm 3.802\times 10^{-2}$	0.349	0.727
taskConditionTMT	$-6.592\times 10^{-3}\pm 3.802\times 10^{-2}$	−0.173	0.862
mechanismCPA2	$6.634\times 10^{-2}\pm 4.657\times 10^{-2}$	1.425	0.154
mechanismCPA3	$1.413\times 10^{-1}\pm 4.657\times 10^{-2}$	3.034	$2.430\times 10^{-3}$
CoM → CoP	$1.812\times 10^{0}\pm 4.657\times 10^{-2}$	38.908	$<2\times 10^{-16}$
axisAP	$-1.354\times 10^{-2}\pm 4.657\times 10^{-2}$	−0.291	0.771
supportConditionWB:mechanismCPA2	$6.269\times 10^{-2}\pm 5.377\times 10^{-2}$	1.166	0.244
supportConditionWB:mechanismCPA3	$8.494\times 10^{-2}\pm 5.377\times 10^{-2}$	1.580	0.114
taskConditionTMT:mechanismCPA2	$-1.183\times 10^{-4}\pm 5.377\times 10^{-2}$	−0.002	0.998
taskConditionTMT:mechanismCPA3	$-6.512\times 10^{-3}\pm 5.377\times 10^{-2}$	−0.121	0.904
supportConditionWB:CoM → CoP	$-1.158\times 10^{0}\pm 5.377\times 10^{-2}$	−21.540	$<2\times 10^{-16}$
taskConditionTMT:CoM → CoP	$-9.096\times 10^{-2}\pm 5.377\times 10^{-2}$	−1.692	0.091
mechanismCPA2:CoM → CoP	$1.138\times 10^{0}\pm 6.586\times 10^{-2}$	17.281	$<2\times 10^{-16}$
mechanismCPA3:CoM → CoP	$8.611\times 10^{-1}\pm 6.586\times 10^{-2}$	13.074	$<2\times 10^{-16}$
supportConditionWB:axisAP	$-1.371\times 10^{-3}\pm 5.377\times 10^{-2}$	−0.026	0.980
taskConditionTMT:axisAP	$5.461\times 10^{-3}\pm 5.377\times 10^{-2}$	0.102	0.919
mechanismCPA2:axisAP	$3.667\times 10^{-3}\pm 6.586\times 10^{-2}$	0.056	0.956
mechanismCPA3:axisAP	$3.685\times 10^{-3}\pm 6.586\times 10^{-2}$	0.056	0.955
CoM → CoP:axisAP	$-3.116\times 10^{-1}\pm 6.586\times 10^{-2}$	−4.732	$2.290\times 10^{-6}$
supportConditionWB:mechanismCPA2:CoM → CoP	$-5.851\times 10^{-1}\pm 7.605\times 10^{-2}$	−7.694	$1.740\times 10^{-14}$
supportConditionWB:mechanismCPA3:CoM → CoP	$-3.858\times 10^{-1}\pm 7.605\times 10^{-2}$	−5.073	$4.060\times 10^{-7}$
taskConditionTMT:mechanismCPA2:CoM → CoP	$1.975\times 10^{-1}\pm 7.605\times 10^{-2}$	2.597	$9.420\times 10^{-3}$
taskConditionTMT:mechanismCPA3:CoM → CoP	$2.307\times 10^{-1}\pm 7.605\times 10^{-2}$	3.033	$2.430\times 10^{-3}$
supportConditionWB:mechanismCPA2:axisAP	$-3.296\times 10^{-2}\pm 7.605\times 10^{-2}$	−0.433	0.665
supportConditionWB:mechanismCPA3:axisAP	$-6.042\times 10^{-2}\pm 7.605\times 10^{-2}$	−0.795	0.427
taskConditionTMT:mechanismCPA2:axisAP	$-2.825\times 10^{-3}\pm 7.605\times 10^{-2}$	−0.037	0.970
taskConditionTMT:mechanismCPA3:axisAP	$7.114\times 10^{-4}\pm 7.605\times 10^{-2}$	0.009	0.993
supportConditionWB:CoM → CoP:axisAP	$9.511\times 10^{-1}\pm 7.605\times 10^{-2}$	12.507	$<2\times 10^{-16}$
taskConditionTMT:CoM → CoP:axisAP	$-9.821\times 10^{-3}\pm 7.605\times 10^{-2}$	−0.129	0.897
mechanismCPA2:CoM → CoP:axisAP	$-6.442\times 10^{-1}\pm 9.314\times 10^{-2}$	−6.916	$5.270\times 10^{-12}$
mechanismCPA3:CoM → CoP:axisAP	$-7.195\times 10^{-1}\pm 9.314\times 10^{-2}$	−7.725	$1.370\times 10^{-14}$
supportConditionWB:mechanismCPA2:CoM → CoP:axisAP	$5.337\times 10^{-1}\pm 1.075\times 10^{-1}$	4.963	$7.200\times 10^{-7}$
supportConditionWB:mechanismCPA3:CoM → CoP:axisAP	$4.617\times 10^{-1}\pm 1.075\times 10^{-1}$	4.293	$1.800\times 10^{-5}$
taskConditionTMT:mechanismCPA2:CoM → CoP:axisAP	$-1.968\times 10^{-1}\pm 1.075\times 10^{-1}$	−1.830	0.068
taskConditionTMT:mechanismCPA3:CoM → CoP:axisAP	$-2.030\times 10^{-1}\pm 1.075\times 10^{-1}$	−1.888	0.059

[1] $\hat{\beta }∼\mathrm{trial}+\left(\mathrm{supportCondition}*\mathrm{taskCondition}\right)*\mathrm{mechanism}*\mathrm{directionality}*\mathrm{axis}+\left(1|\mathrm{participant}\right)$. [2] Boldfaced values indicate statistical significance at p<0.05.

## Data Availability

The dataset used in this study can be obtained from the corresponding author, Madhur Mangalam (mmangalam@unomaha.edu), upon reasonable request.
